# Potential Defence Mechanisms Triggered by Monosodium Glutamate Sub-Chronic Consumption in Two-Year-Old Wistar Rats

**DOI:** 10.3390/nu15204436

**Published:** 2023-10-19

**Authors:** Octavia-Laura Moldovan, Camil-Eugen Vari, Amelia Tero-Vescan, Ovidiu Simion Cotoi, Iuliu Gabriel Cocuz, Flaviu Alexandru Tabaran, Romelia Pop, Ibolya Fülöp, Rafael Florin Chis, Ioana-Andreea Lungu, Aura Rusu

**Affiliations:** 1 Medicine and Pharmacy Doctoral School, George Emil Palade University of Medicine, Pharmacy, Science, and Technology of Targu Mures, 540142 Targu Mures, Romania; ioana-andreea.lungu@umfst.ro; 2 Pharmacology and Clinical Pharmacy Department, Faculty of Pharmacy, George Emil Palade University of Medicine, Pharmacy, Science, and Technology of Targu Mures, 540142 Targu Mures, Romania; camil.vari@umfst.ro; 3 Medical Chemistry and Biochemistry Department, Faculty of Medicine in English, George Emil Palade University of Medicine, Pharmacy, Science, and Technology of Targu Mures, 540142 Targu Mures, Romania; amelia.tero-vescan@umfst.ro; 4 Pathophysiology Department, Faculty of Medicine, George Emil Palade University of Medicine, Pharmacy, Science, and Technology of Targu Mures, 540142 Targu Mures, Romania; ovidiu.cotoi@umfst.ro (O.S.C.); iuliu.cocuz@umfst.ro (I.G.C.); 5 Pathology Department, Mures Clinical County Hospital, 540011 Targu Mures, Romania; 6 Department of Pathology, Faculty of Veterinary Medicine, University of Agricultural Sciences and Veterinary Medicine of Cluj-Napoca, 400372 Cluj-Napoca, Romania; alexandru.tabaran@usamvcluj.ro (F.A.T.); romelia.pop@usamvcluj.ro (R.P.); 7 Toxicology and Biopharmacy Department, Faculty of Pharmacy, George Emil Palade University of Medicine, Pharmacy, Science, and Technology of Targu Mures, 540142 Targu Mures, Romania; ibolya.fulop@umfst.ro; 8 Faculty of Medicine, George Emil Palade University of Medicine, Pharmacy, Science, and Technology of Targu Mures, 540142 Targu Mures, Romania; chisrafael98@gmail.com; 9 Pharmaceutical and Therapeutic Chemistry Department, Faculty of Pharmacy, George Emil Palade University of Medicine, Pharmacy, Science, and Technology of Targu Mures, 540142 Targu Mures, Romania; aura.rusu@umfst.ro

**Keywords:** glutamic acid, monosodium glutamate, sub-chronic toxicity, oxidative stress, morphological analysis, biochemical parameters

## Abstract

Monosodium glutamate (MSG) is the sodium salt of glutamic acid (GLA), used as a flavour enhancer. MSG is considered a controversial substance. It is incriminated in disturbing the antioxidant system, but also has beneficial effects, as GLA metabolism plays a crucial role in homeostasis. This study highlights which positive or negative aspects of MSG sub-chronic consumption are better reflected in subjects potentially affected by advanced age. Daily doses of MSG were administered to four groups of two-year-old Wistar rats for 90 days: (I) 185 mg/kg bw, (II) 1500 mg/kg bw, (III) 3000 mg/kg bw and (IV) 6000 mg/kg bw, compared to a MSG non-consumer group. Aspartate aminotransferase, alanine aminotransferase, alkaline phosphatase, direct and total bilirubin, total cholesterol, triglycerides, creatinine and urea levels were analysed; stomach, liver and kidney samples were subjected to histopathological analysis. Although, in most cases, there were no statistical differences, interesting aspects of the dose–effect relationship were observed. After MSG sub-chronic consumption, the positive aspects of GLA seem to be reflected better than the negative ones. The hormesis effect, with low-level reactive oxygen species’ protective effects and GLA metabolism, may represent the hypothesis of a potential defence mechanism triggered by MSG sub-chronic consumption in ageing rats.

## 1. Introduction

Glutamic acid (GLA) is a non-essential amino acid found in animal and plant proteins [1]. It is a compound of great importance for human homeostasis, with implications for the metabolism of proteins, lipids and carbohydrates. Also, it is a component of the antioxidant glutathione and a major excitatory neurotransmitter in the human and mammalian brains [2]. Monosodium glutamate (MSG) is the sodium salt of GLA, with flavour enhancing properties [1]. MSG is widely used in many food ingredients to promote palatability [3]. MSG is responsible for the fifth sense, called umami, and it is classified as a food additive by the food safety regulatory agencies (E 621) [1,4]. Also, MSG can be an alternative to increase salivary secretion and appetite. It can improve the palatability of food with low fat and salt content, reducing the intake of salt in food. Nowadays, MSG is produced by fermenting starch, sugar beet, sugar cane or molasses, mainly using bacteria of the genus *Corynebacterium* [1,5]. The umami taste is attributed to the glutamate ion [1]. There is no difference between the glutamate and MSG forms in terms of processes in the organism. Glutamate is absorbed from the intestinal lumen and then widely metabolised by enterocytes through oxidation [4]. Only a minimal quantity is subsequently found in the portal blood [6,7]. We previously presented the biological role of GLA and its metabolic implications [8].

In 1995, MSG was included in the Generally Recognised As Safe (GRAS) list by the Food and Drug Administration (FDA) [4,6]. Also, the Joint Food and Agriculture Organization for the United Nations/World Health Organization (FAO/WHO) Expert Committee on Food Additives has assessed the overall safety of MSG. As a result, the food additive has been labelled safe without presenting any risk or danger to health [1]. The European Food Safety Authority (EFSA) also reassessed the safe amount of MSG as a food additive over time. According to them, in 2017, the acceptable daily intake (ADI) was 30 mg MSG/kg body weight per day, expressed as GLA, for GLA and GLA salts (E 620–E 625). This value was established based on the No observed adverse effect level (NOAEL) of 3200 mg MSG/kg body weight per day (identified from a neurodevelopmental study) [9]. The producers are limited to adding GLA or its salts up to 10 g/kg or 10 g/L of food. Instead, E 620–E 625 are authorised at quantum satis in salt substitutes, seasonings and condiments [1,4,9]. The problem is the increasing MSG usage in processed food, mostly without labelling the added quantity. Thus, the daily consumption of MSG cannot be accurately calculated [3]. Also, the daily consumption of MSG is influenced by the multiple sources of glutamate intake. It may come from its natural occurrence as a constituent of proteins, from free glutamate in certain fermented foods and the addition of glutamic acid and glutamates to food as additives [10]. It is considered that, even without added glutamate, for most age categories, consumption of glutamate exceeds the 30 mg/kg bw/day ADI established by the EFSA [11]. The average daily consumption in European countries seems to be around 0.3–1.0 g/day, exceeding 1.0 g/day in Asia [4,12]. Details about international regulations regarding MSG consumption are given by Moldovan O. et al. (2021) [8]. 

Although food safety regulatory agencies generally consider MSG consumption safe, there are two aspects regarding its safety as a food additive. On one side, using GLA and its salts as food additives makes a small contribution to the total glutamate intake from all sources [10]. Also, it is considered that exogenous glutamate does not passively cross the cellular membranes, being metabolised as an energy substrate in the small intestine [13]. Conversely, studies providing evidence of MSG’s toxic effects have raised questions about MSG intake as a flavour enhancer [2]. Therefore, using MSG as a food additive is controversial [5]. 

### Positive and Negative Aspects of the Biological Behaviour of MSG

Because MSG follows the same path of processing as GLA by the human organism, some essential metabolic roles of the amino acid highlight their beneficial effect. Firstly, GLA is involved in liver detoxification because of its capacity to fix the ammonia in case of acute liver failure. Physiologically, intestinal ammonia, produced from nitrogen products, is taken up by the liver and subjected to the ureogenetic cycle. Damaged livers, instead, do not accomplish this step efficiently, resulting in high ammonia levels. The glutamine (GLN)/glutamate cycle and glutamine synthetase (GS) are very important in the process of ammonia detoxification, as GLN is the non-toxic storage and transport form of ammonia in the liver [14]. The enzyme catalyses GLN formation from GLA and ammonia, the reaction taking place in almost all tissues in the body [15]. This biochemical reaction is also significant at the cerebral level, as 90% of hepatic disorders are accompanied by increased brain ammonia. It is a major neurotoxin involved in hepatic encephalopathy. The astrocytic GS metabolises ammonia, resulting in elevated plasma and brain GLN [16,17].

The GLA–GLN relationship also plays a vital role in muscular metabolism, as GLN is stored and released predominantly by the skeletal muscle [18]. Also, it is the most abundant non-essential amino acid in human muscle [19]. The skeletal muscle amino acid metabolism generates GLN to detoxify the ammonia produced at this level [18]. Because of its immunomodulatory role, GLN is widely used in sports nutrition as a food supplement [20]. An experimental study concluded that GLN supplementation increases muscular power and strength and enhances performance during resistance training [19]. Also, sports recovery acceleration and muscular fatigue reduction are essential for sports performance. In this regard, glutamine supplementation seems to increase muscle glycogen synthesis and reduce ammonia accumulation induced by exercise; it may attenuate markers of muscle damage, such as blood creatine kinase and lactate dehydrogenase levels; it can increase glutathione synthesis, being considered an indirect antioxidant [20,21].

In addition to being the major nitrogen transporter, GLN is also a regulator of acid–base balance [18]. Kidney-type glutaminase is a hydrolase that generates GLA and ammonia from GLN, and it is increased only in the kidney in response to metabolic acidosis [22]. The primary source of ammonia is GLN, which gets excreted in the urine. The acidotic condition stimulates the delivery and transport of glutamine into the kidney. When the body is exposed to an acid environment, the production of ammonia and its excretion are major mechanisms by which the kidney produces bicarbonate [15].

Additionally, the contribution of GLA to synaptic transmission, neuronal plasticity, development, learning and memory is well established [23]. In contrast, with γ-aminobutyric acid (GABA), an inhibitor neurotransmitter in the central nervous system, glutamate controls excitatory neurotransmission [24]. NMDA (*N*-methyl-D-aspartate) receptors of glutamate are considered to be of great importance for the learning process. They seem to have a crucial role in encoding. NMDA receptors are involved in the emotional memory, the procedural memory system, motor learning and episodic-like memory [23]. In addition, group I of metabotropic receptors of glutamate (mGluRs) is involved in learning and long-term memory formation of hippocampal formation. Hippocampal mGlu5 receptor has a unique role in memory processing [24].

On the other hand, despite GLA metabolic implications and beneficial effects, many researchers disagree with the safety of MSG consumption. The potential toxicity of MSG has been evaluated mainly through preclinical studies. The reported side effects of MSG included (but were not limited to) hepatotoxicity, nephrotoxicity, reproductive system toxicity, cardiotoxicity, neurotoxicity [25,26,27,28,29,30,31]. It has been demonstrated that administration of MSG resulted in histological alterations and impairment in biochemical and metabolic markers such as aspartate aminotransferase, alanine aminotransferase, alkaline phosphatase, total or direct bilirubin [32,33], cholesterol, triglycerides, creatinine [34] and urea [35].

**Figure 1:**
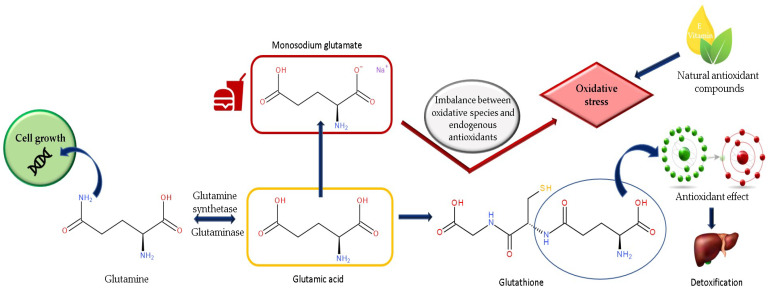
Biochemical relationship between glutamic acid, glutamine and glutathione. MSG’s most likely mechanism for producing cytotoxicity.

Oxidative stress is considered the primary mechanism for many disorders caused by MSG [4]. An imbalance between oxidative species and endogenous antioxidant compounds could be the mechanism of MSG toxicity. Excessive stimulation of MSG receptors alters calcium homeostasis and initiates free radical formation, mitochondrial dysfunction and apoptosis [1]. The liver is the main organ that detoxifies the body. When the liver undergoes morphological changes, the metabolic processes are affected. Thus, the affected metabolic processes alter the detoxification process [36]. We can link an alteration in the redox potential with an increase in lipid peroxidation processes [37,38]. Therefore, it is considered that MSG consumption could be associated with the production of reactive oxygen species (ROS) and, thus, lipid peroxidation and inflammation in several tissues, such as the liver, kidney and brain [38,39]. Experimental studies demonstrate that the use of MSG has been linked to decreased levels of glutathione (GSH), glutathione S transferase (GST), superoxide dismutase (SOD) and catalase (CAT) [33,37]. Also, this idea is supported by the potential of some natural antioxidant compounds to reduce the alterations caused by MSG at different histological levels. Among these substances are vitamin E, curcumin, *Terminalia catappa* extract, propolis and protocatechuic acid [29,36,40,41,42] (Figure 1). We previously presented the protective compounds and their effects against various MSG toxicities [8]. 

Therefore, questions can be raised about the consumer’s dangerous and unconscious abuse of MSG. The reason is the difficulty of monitoring the amount of ingested substance, which is likely responsible for effects at multiple histological levels in chronic and sub-chronic consumption, even in small doses. The scientific literature provides us with several experimental studies that have assessed the chronic toxicity of MSG but with significant heterogeneity in the administered doses and the period of administration. Most preclinical studies evaluate MSG chronic and sub-chronic toxicity at different tissue levels through biochemical and/or histological determinations (Table 1).

Therefore, this experimental study aims for a more comprehensive knowledge of MSG’s pharmaco-toxicological profile, considering its abusive and uncontrolled use in food. As presented before, arguments exist for and against its behaviour in the organism. This study emphasises which positive or negative aspects of MSG sub-chronic consumption are better highlighted in subjects potentially affected by advanced age; this aspect involves observing how they are influenced by the ADI and NOAEL dose of MSG, established by the scientific literature. Thus, the study goal is to provide relevant data regarding the effect of MSG through biochemical and histological methods (hepatic, stomach and renal tissues) applied to an ageing experimental animal model (two-year-old Wistar rat).

## 2. Materials and Methods

### 2.1. Approvals

The study proposal was approved by the George Emil Palade University of Medicine, Pharmacy, Science, and Technology of Targu Mures Scientific Research Ethics Committee (No. 1172/05.11.2020) and Targu Mures Sanitary-Veterinary and Food Safety Direction (No. 49/02.07.2021).

### 2.2. The Used Chemical Nutrient

Anhydrous MSG was obtained from Sigma Aldrich (CAS: 142-47-2; Code: 102366233; Batch: BCCF0636). The producer guaranteed 99% purity.

### 2.3. Experimental Design

#### 2.3.1. Animals and the Experimental Conditions

The study was conducted on seventy 2-year-old Wistar adult rats (females and males). They were obtained from the Biobase of UMFST George Emil Palade of Targu Mures. The animals were raised under standard laboratory conditions: comfort temperature 21–22 °C, humidity 40–60%, 12 h/12 h dark–light cycle. The animals were divided into five groups of 14 rats each (10 females and 4 males/group). The animals were fed with standard fodder for rats purchased from “Cantacuzino” National Institute for Medical-Military Research and Development from Bucharest. The MSG was included in the feed composition, and the mixture was homogenised and pelleted. The administration of the MSG to the subjects was carried out under identical conditions for 90 days.

#### 2.3.2. Self-Administration of MSG in Rats

The pellets and water were administered ad libitum, as this feeding method is often used for rodents in animal experiments and is considered a stress-free administration route. Additionally, as in the human population MSG is administered orally, we considered it more relevant for the study to choose this route to simulate human behaviour as correctly as possible. 

#### 2.3.3. The Doses Used in the Animal Model Experiment

The administered amounts were established considering the quantities allowed by the food safety authorities (ADI and NOAEL doses according to EFSA) since the ones used in similar studies are very different. The MSG doses per group and pertinent observations are presented in Table 2.

Human/animal and animal/human dose extrapolation were set after Reagan-Shaw S. et al. [46] and Anroop B. Nair et al. [47].

#### 2.3.4. Measurements during the Study 

The groups were monitored daily regarding their behaviour and experimental conditions. Also, the rats were weighed, and the food and water consumed were monitored weekly.

#### 2.3.5. Biological Tests 

After 90 days of MSG administration, the animals were sacrificed (following an 18 h fast) to collect the biological samples—blood samples (blood serum) and histological samples. The blood samples were used to determine the levels of several biochemical and metabolic parameters: aspartate aminotransferase (AST), alanine aminotransferase (ALT), alkaline phosphatase (ALP), total bilirubin (TB) and direct bilirubin (DB), urea (UR), creatinine (CR), triglycerides (TG) and total cholesterol (CHOL). Moreover, liver, renal and stomach tissues were collected for anatomopathological analysis.

Application of anaesthesia. The blood samples were collected through cardiac puncture under general gaseous anaesthesia using isoflurane. The exsanguination allowed the collection of a high amount of blood to analyse all the proposed biochemical parameters. 

Sampling and storage of blood samples. The blood samples were collected using a 10 mL or 20 mL syringe. They were collected in 6 mL yellow vacuum blood sample collection tubes (Gel and Clot activator) for optimal separation of blood serum. Then, the samples were kept for 30 to 50 min to rest at room temperature. Further, they were centrifuged for 5 min at 3000 rotations/min, using 5804 R Eppendorf and 5810 Eppendorf centrifuges. After centrifugation, the serum was separated from the other components using an automatic micropipette and stored in safe-lock Eppendorf tubes at −80 °C.

The analysis of biochemical and metabolic parameters. The analysis of the parameters was made with Cobas Integra 400 Plus apparatus. The kits were compatible with the analyser and were purchased from Roche Diagnostics. The parameters were analysed with specific methods and reagents (Table 3).

Sampling and storage of the tissue samples. The liver, kidneys and stomach were collected, rinsed and conserved in a 10% neutral buffered formalin (NBF). A particular procedure was applied to the stomach, which was rinsed with 9% NaCl solution and left on a Petri dish covered with 10% NBF for 30 min. After that, the tissue was conserved in a 10% NBF. After 24 h, the solution was changed for all the samples. Then, the tissue samples were subjected to grossing and inclusion in histological cassettes. The tissue samples were processed following a standard histological protocol and embedded in paraffin. After this, thin sections of 2 µm were obtained from the paraffin blocks using the rotary microtome. They were later stained using Haematoxylin–Eosine (H&E), according to a routine protocol.

Histological analysis. Histological samples were examined under an Olympus BX51 microscope, and the bright field images were obtained with an Olympus SP350 digital camera and processed using the Olympus cellSens software (Version 3.1). Histologic changes were assessed and graded as to severity utilising the International Harmonization of Nomenclature and Diagnostic (INHAND) standards [48,49,50,51,52,53,54]. The microscopic changes were graded according to severity, utilising a standard grading system whereby 0 = no significant change, 1 = minimal, 2 = mild, 3 = moderate and 4 = severe. Chronic progressive nephropathy (CPN) was also graded to establish the severity. 

### 2.4. Statistical Analysis of the Results

The data were collected and stored with the help of Microsoft Excel (Microsoft Office™) software (Office 365). The statistical tests were performed in Graphpad PRISM 9 (Graphpad™). All quantitative data underwent the ROUT (Q = 1%) outlier identifier test. The normality distribution was tested with the D’Agostino and Pearson test. To determine whether the experimental groups had any statistical differences regarding the biochemical and metabolic parameters and chronic progressive nephropathy severity, the one-way ANOVA test and post-hoc Dunnett’s and Tukey’s tests were used for statistical analysis. Data are expressed as mean values ± SD. All statistical tests were performed at a 95% confidence interval. Statistical significance was established, being indicated by *p* < 0.05. 

In the case of histopathological data, no statistical tests were applied. The results are expressed as the occurrence frequency of modification/group. 

## 3. Results

### 3.1. Biochemical and Metabolic Parameters 

#### 3.1.1. Aspartate Aminotransferase (AST) and Alanine Aminotransferase (ALT)

**Figure 2:**
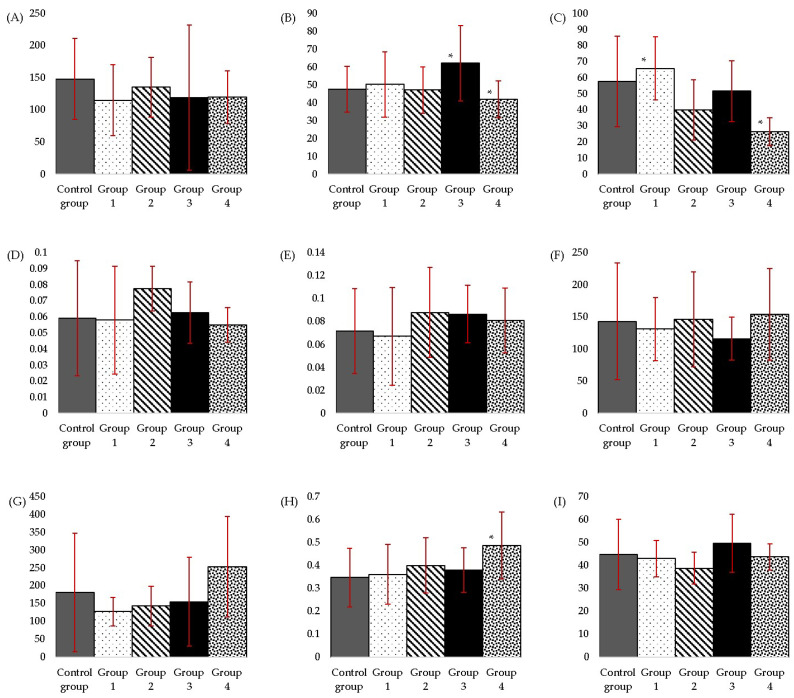
Graphical representation of (**A**) aspartate aminotransferase, (**B**) alanine aminotransferase, (**C**) alkaline phosphatase, (**D**) direct bilirubin, (**E**) total bilirubin, (**F**) total cholesterol, (**G**) triglycerides, (**H**) creatinine, (**I**) urea values measured after three months of daily MSG administration in two-year-old rats. Values represent the mean ± SD. One-way ANOVA, post-hoc, Dunnett and Tukey’s tests were performed (*p* ≤ 0.05). Considering: a: ALT, a statistical difference was observed between groups 3 and 4; b: ALP, a statistical difference was observed between the Control group and group 4 and between group 1 and group 4; c: CR, a statistical difference was observed between the Control group and group 4 (statistical differences being marked with *).

No statistical differences were observed between the groups for the AST parameter. The highest values for AST were observed in the Control group, followed by group 2. For ALT, the highest values were found in group 3; a decrease in values of ALT from group 3 to group 4 was observed, with a statistically significant difference (Figure 2; Table S1 in Supplementary Materials).

#### 3.1.2. Alkaline Phosphatase (ALP)

In the present study, group 4 presents the lowest values of ALP. Group 1 shows the highest values, followed by the Control group. Statistical differences were observed between group 1 and group 4 and also between the Control group and group 4 (Figure 2; Table S2 in Supplementary Materials).

#### 3.1.3. Direct Bilirubin (DB) and Total Bilirubin (TB)

The highest values of DB and TB were observed in group 2. A decrease in the values of DB (slightly decrease) and TB from the Control group to group 1 was noticed. Also, a decrease of TB (slightly decrease) and DB from group 2 to groups 3 and 4 was observed (Figure 2; Table S3 in Supplementary Materials); there are no statistical differences between the groups. 

#### 3.1.4. Total Cholesterol (CHOL)

The maximum CHOL value was measured in group 4, which received the highest dose, twice the NOAEL-approved one. The differences between the groups were not statistically relevant regarding CHOL levels. Group 2 had similar values to the Control group. Group 3 presented the lowest values compared to the rest (Figure 2; Table S4 in Supplementary Materials).

#### 3.1.5. Triglycerides (TG)

TG levels increased from group 1 to group 4; the last one showed the highest values among all five groups. Groups 1, 2 and 3, on the other hand, showed lower values than those of the Control group (Figure 2; Table S5 in Supplementary Materials). 

#### 3.1.6. Creatinine (CR)

The Control group and groups 1 to 3 presented similar values of CR. The highest values were those of group 4; a statistical difference was observed between the Control group and group 4 (Figure 2; Table S6 in Supplementary Materials).

#### 3.1.7. Urea (UR)

The measured values of UR were quite similar for all groups; the highest value was observed in group 3 (Figure 2; Table S7 in Supplementary Materials).

### 3.2. Histopathological Analysis

**Figure 3:**
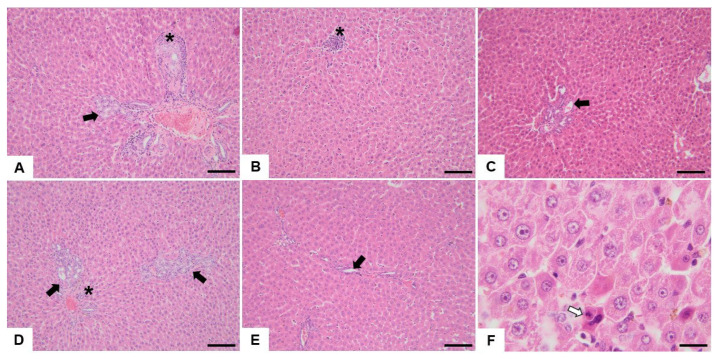
Histopathological images of the liver from the Control and experimental groups (image (**A**)—Control, image (**B**)—group 1, image (**C**)—group 2, image (**D**)—group 3, and images (**E**) and (**F**)—group 4). Multifocally, moderate biliary duct hyperplasia (indicated by black arrows) is associated with inflammatory mononuclear cell infiltrates (indicated by the asterisks). Multifocally, there is necrosis involving isolated hepatocytes (‘‘single cell necrosis’’) (image (**F**), indicated by the white arrow). Scale bar = 100 µm (images (**A**–**E**)) and = 20 for image (**F**). H&E stain, objective ×20 (images (**A**–**E**)) and ob ×100 (image (**F**)).

**Figure 4:**
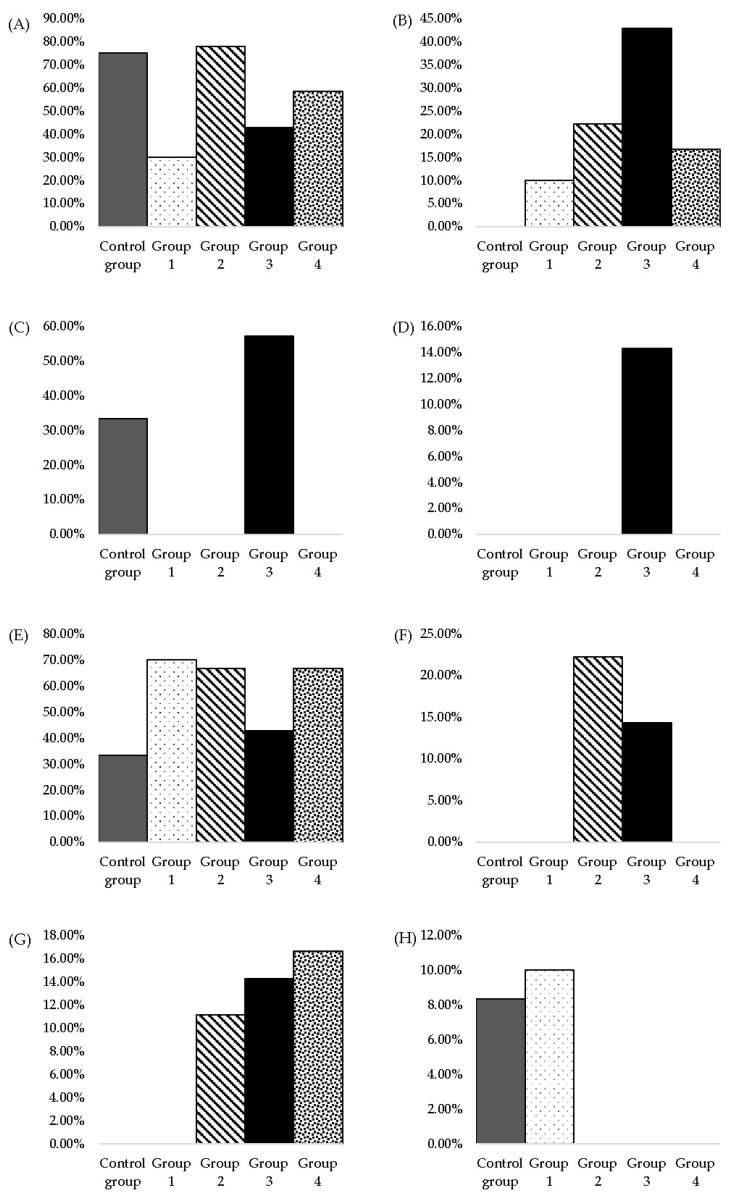
Histopathological modifications of hepatic tissue measured after three months of daily MSG administration in two-year-old rats: (**A**) minimal bile-duct hyperplasia, (**B**) moderate bile-duct hyperplasia, (**C**) minimal oval cell hyperplasia, (**D**) moderate oval cell hyperplasia, (**E**) minimal multifocal mononuclear cell infiltrate, (**F**) moderate multifocal mononuclear cell infiltrate, (**G**) minimal hepatic focal microvesicular steatosis, (**H**) minimal glycogenosis.

The histological changes observed in the hepatic tissue are presented in Table 4, Figure 3 and Figure 4, expressed as the frequency of modification depending on the dose administered to each group.

**Figure 5:**
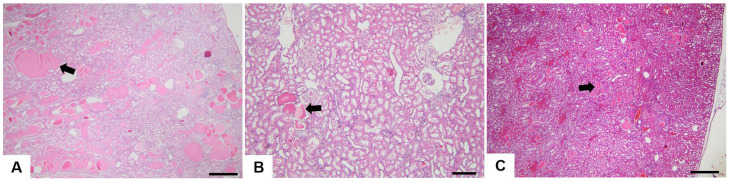
Histopathological images of the kidney from the Control and experimental groups (image (**A**)—Control, image (**B**)—group 1, image (**C**)—group 3). Multifocally, there are hyaline casts (indicated by the black arrows) with prominent renal tubule dilation (indicative of chronic progressive nephropathy). Scale bar = 400 µm (images (**A**,**C**)) and 150 µm (image (**B**)). H&E stain, objective ×4 (images (**A**,**C**)) and ×10 (image (**B**)).

**Figure 6:**
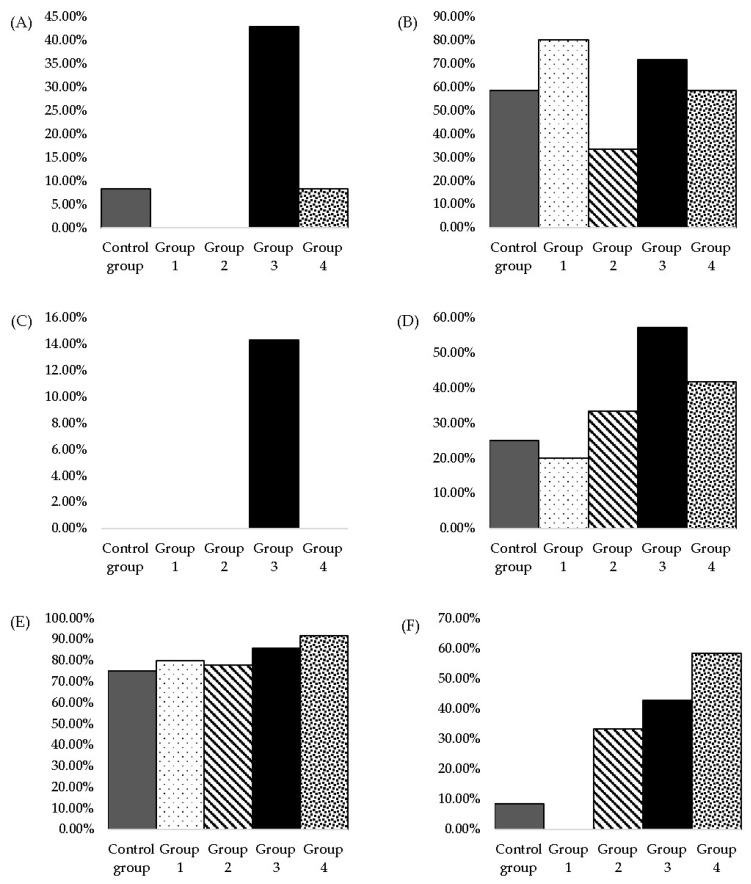
Histopathological modifications of renal tissue measured after three months of daily MSG administration in two-year-old rats: (**A**) hyaline droplet accumulation, (**B**) tubular pigment, (**C**) mineralisation, (**D**) glomerulopathy, (**E**) chronic progressive nephropathy, (**F**) cytoplasmic vacuolisation.

The histological changes of the renal tissue are also presented as the occurrence frequency of lesions for each group. Hyaline droplet accumulation, tubular pigment, mineralisation, chronic progressive nephropathy, glomerulopathy and cytoplasmic vacuolisation are the histopathological changes observed among the subjects. (Table 5, Figure 5 and Figure 6).

As a characteristic of CPN, glomerulopathy was observed in all groups, as follows: Control group—25.00% (3/12); group 1—20.00% (2/10); group 2—33.33% (3/9); group 3—57.14% (4/7); group 4—41.66% (5/12).

**Figure 7:**
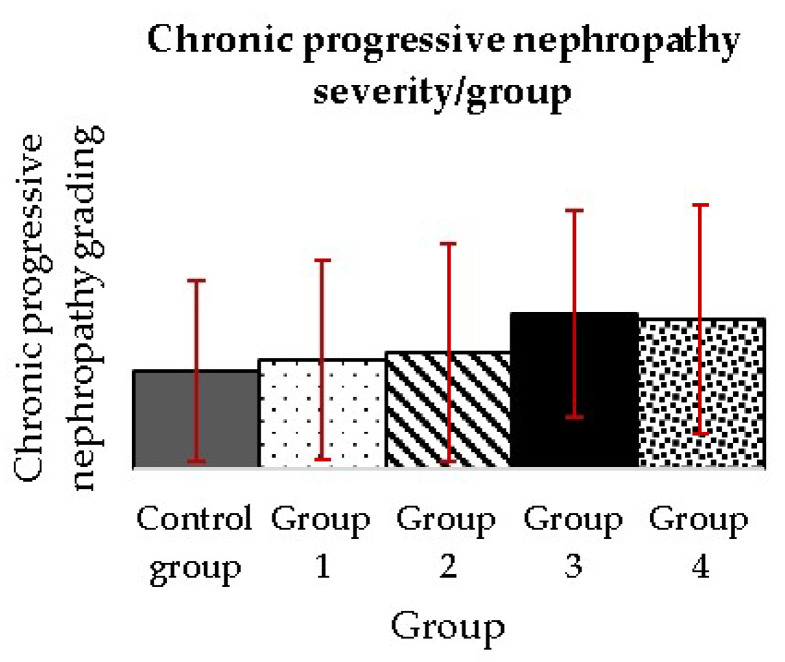
Chronic progressive nephropathy grade measured after three months of daily MSG administration in two-year-old rats. Values represent the mean ± SD. One-way ANOVA, post-hoc, Dunnett and Tukey’s tests were performed (*p* ≤ 0.05). No statistically significant differences were observed.

Also, a grading of chronic progressive nephropathy was performed to assess the severity of the alteration. No statistical differences were observed between the groups’ grades after applying the one-way ANOVA and post-hoc Dunnett’s and Tukey’s tests (Figure 7; Table S8 in Supplementary Materials). 

### 3.3. Mortality Rate among the Groups

**Figure 8:**
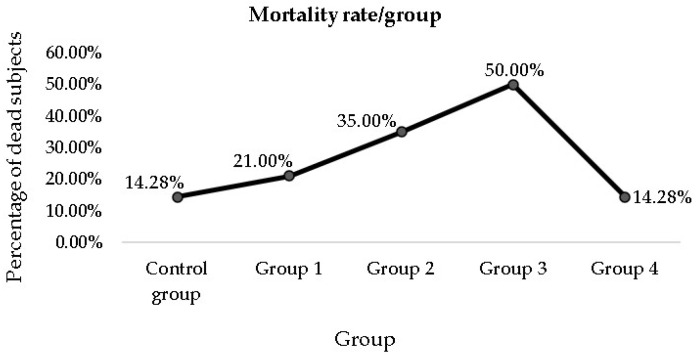
The mortality rate among the experimental and Control groups (expressed as a percentage of the total number of subjects/group).

Throughout the experiment, some subjects from each group died before the end of the administration of the entire amount of MSG. The mortality rate increased from the Control group to group 3, with a subsequent decrease in group 4 (mortality rate expressed as a percentage of the total number of subjects/group) (Figure 8).

## 4. Discussion

### 4.1. Analysis of Changes in Biochemical Parameters

#### 4.1.1. Aspartate Aminotransferase (AST) and Alanine Aminotransferase (ALT) Changes

AST and ALT are indicators of hepatocyte integrity, an increase in the serum levels of the two enzymes suggesting the destruction of the cytoplasmic or mitochondrial membranes. Increased release of the liver enzymes AST and ALT indicates hepatocellular damage in mammals. For rodents, AST shows greater specificity for liver damage [55]. 

AST and ALT are transaminases that can transform glutamate into α-ketoglutarate (α-KG). Once in the intestine, exogenous glutamate undergoes this transformation catalysed by AST and ALT in the intestinal enterocytes. The final product is carbon dioxide after participating in this way in the Krebs cycle. However, glutamate metabolism also yields glutamine, aspartate or alanine [31,56].

Various scientific studies present MSG as responsible for destroying liver cells by producing an imbalance between the body’s oxidising and antioxidant species. The explanation could be some disturbances in the levels of CAT and SOD or the decrease in the level of GSH in the case of high consumption of MSG [31,42,56,57]. Thus, MSG’s cytotoxic effect results from oxidative stress production, damaged liver cells and canaliculi and subsequent release of enzymes into the circulation [44]. 

Scientific studies that evaluated the toxicity of MSG show relatively significant heterogeneity in terms of the administration period and the administered dose. In most studies, the administration was performed orally with the feed or by gavage. Studies conducted on young adult rats concluded that different amounts of MSG are responsible for increasing AST and ALT levels. Also, in studies with multiple doses, the increase of AST and ALT was directly proportional to the administered dose. MSG had been given in different amounts and for different periods and in each case, the AST and ALT levels were higher than the Control group: 0.69 mg/g bw/day and 1.38 mg/g bw/day, respectively, administered for 28 days [58]; 1.8 mg/kg bw/day, for 30 days [59]; 4 mg/kg bw/day were administered for 14 [38] and seven days [42], respectively, and increased levels of AST and ALT were observed in both cases; 5 mg/kg bw/day, for 4 weeks [37]; 10 mg/kg bw/day, for 30 and 60 days, respectively [36]; 35 mg/kg bw/day, for 14 days [60]; 50 mg/kg bw/day, for 4 weeks [57]; 97 mg/kg bw/day, for 6 weeks [43]; 120 mg/kg bw/day, for 3 months; 600 mg/kg bw/day, for 28 days [61]. Also, several studies administered MSG in doses between 2 and 8 g, similar to those that had been given in our research to groups 3 and 4. Increased levels of both enzymes were observed compared to their Control group: 2 g/kg bw/day, for 4 weeks [25,29] and 2.4 g/kg bw/day, for 8 weeks [33]; 4 g/kg bw/day, for 14 days [62]; 5 g/kg bw/day, for 30 days [35]; 8 g/kg corp, for 14 days [63]. Some studies mentioned above used different products, especially natural ones, to counterbalance MSG toxicity. 

In our study, compared with the findings of the studies mentioned above, the values of AST and ALT are not proportional to the administered dose. We observed a decrease in values from group 3 to group 4 for ALT. Although the quantities of MSG administered in our study are much higher than in other studies, there are no statistical differences between the groups, except groups 3 and 4, for the ALT parameter.

#### 4.1.2. Alkaline Phosphatase (ALP)

The relevance of the clinical value of serum ALP lies in diagnosing cholestatic liver, with hepatobiliary obstruction being a pathological condition in which it is increased [38,64]. Given that cholestatic syndrome can have an extrahepatic or intrahepatic origin, we considered that the enzyme might be relevant for evaluating MSG liver toxicity. Similar to AST and ALT, the more significant the degree of hepatocyte damage, the higher the extravasation of the enzyme into the bloodstream [35]. 

Several experimental studies on rats presented MSG as the responsible molecule for increased levels of ALP. An increase in ALP levels compared to the Control group was observed, even if there was a difference in the administered dose and the period of administration, such as: 2.4 g/kg bw/day, 8 weeks [33]; 35 mg/kg bw/day, 14 days [60]; 600 mg/kg bw/day, 28 days [61]; 1.8 mg/kg bw/day, 30 days [59]; 4 mg/kg bw/day, 14 days [38]; 50 mg/kg bw/day, 4 weeks [57]. 

In our study, group 4 (6 g MSG/kg bw/day) presented the lowest ALP values compared to group 1 (185 mg MSG/bw/day), the differences being statistically significant. Also, statistical differences exist between group 4 and the Control group. The obtained results contrast with the Johnlouis I. et al. (2019) experimental results, where an increase in ALP levels, compared to the Control group, was observed at 8 g MSG/kg bw/day after 14 days of administration [63]. The same result was reported by Al-Mousawi N. H. (2017) (5 g/kg bw/day, 30 days of administration) [35]. In another study, where 5 mg MSG/kg bw/day was administered for four weeks to young male rats (9–10 weeks), no relevant changes were observed after administration of MSG [37]. Two experimental studies in which 4 mg MSG/kg bw/day were administered to adult rats for 21 and 28 days, respectively, concluded that ALP levels where lower after MSG administration, in one case at the cerebral level [65], and in the other one at testicular level [66]. 

#### 4.1.3. Direct Bilirubin (DB) and Total Bilirubin (TB)

Serum bilirubin concentration can be used to indicate liver functional status and differentiate several types of liver damage [67]. An increased level of TB is an indicator of cirrhosis or haemolysis, and both this and DB may indicate biliary or hepatic duct obstruction [68,69,70]. According to scientific literature, MSG induces erythrocyte oxidative haemolysis [71]. The elevated levels of DB, TB and IB could also result from kidney damage caused by MSG [72]. Conjugated bilirubin, once it reaches the intestine, is transforms into urobilinogen. Most of it is eliminated through faeces, but a small part is resorbed in the intestine and excreted through urine. Thus, elevated levels of DB could indicate defective elimination caused by renal dysfunction. 

Onobrudu D. et al. (2020) [38] and Airaodion A. et al. (2020) [72] observed dose-dependent increasing levels of DB after MSG administration. Most preclinical studies regarding MSG’s effect on TB levels indicate an increase of the parameter compared to the Control group: 97 mg/kg bw/day, administered for 6 weeks [43]; 35 mg/kg bw/day, for 14 days [60]. Other studies in which higher doses, similar to the ones we administered, were given to the subjects indicated a dose-dependent increase in TB levels: 2 g/kg bw/day, 4 g/kg bw/day and 8 g/kg bw/day, for 14 days [73]; 2.4 g/kg bw/day, for 8 weeks [33]; 8 g/kg bw/day, for 14 days [63]. In contrast with these findings, our study observed that DB and TB levels did not suffer changes directly proportional to the MSG-administered dose, with group 2 having the highest values for both DB and TB. The increase in MSG dose is correlated with decreased bilirubin levels. The decreased DB from group 2 to group 4 raises questions about the body’s response to high amounts of MSG. As for TB levels, they follow roughly the same downward trend from group 2 to group 4, but the differences are not as pronounced. This type of variation in TB levels is also found in the Airaodion A. et al. (2020) study, where the group with 500 mg/kg bw/day had lower values than the Control group; the group with 750 mg/kg bw/day and 1 g/kg bw/day had increasing values; the group with 1250 mg/kg bw/day had lower values than the group with 1 g/kg bw/day (at eight weeks administration of MSG) [72]. In another study, TB levels decreased in a manner correlated to the increased MSG dose [67].

#### 4.1.4. Total Cholesterol (CHOL)

CHOL and TG are good indicators for the changes in the serum lipid profile following sub-chronic consumption of MSG. Elevated levels of CHOL indicate the onset of atherosclerotic processes, kidney damage or the development of diabetes. Low levels, on the other hand, may suggest liver damage [70]. MSG is closely related to carbohydrate and lipid metabolism through its involvement in the Krebs cycle. Thus, MSG is probably responsible for disrupting optimal blood sugar and insulin levels, potentially leading to insulin resistance syndrome [74,75]. Insufficient insulin levels can cause hypercholesterolemia and hypertriglyceridemia [76]. 

According to the scientific experimental studies on animals that assessed the influence of CHOL according to the dose of MSG, an increase in CHOL levels was predominantly observed compared to MSG non-consuming groups. The values were also directly proportional to the increase in the quantity of the additive. So, starting from the thorough analysis of the experimental studies and considering the metabolic implications of MSG, the expectations regarding CHOL levels were that they would increase depending on the dose of the additive. Several studies present higher levels of CHOL after MSG administration, as follows: 15 mg/kg bw/day, administered for 30 days [77]; 0.69 mg/g bw/day and 1.38 mg/g bw/day, for 28 days [58]; 600 mg/kg bw/day, for 28 days [61]; 200 mg/kg bw/day, for 14 days [28]; 50 mg/kg bw/day, for 4 weeks [57]; 10 mg/kg bw/day, for 30 days and 60 days [36]; 200 mg/kg bw/day, 400 mg/kg bw/day and 600 mg/kg bw/day, for 28 days [30]. It was interesting to observe that, in two different studies, 4 mg/kg bw/day, administered for 14 days [38], respectively 7 days [42], to adult rats produced increased levels of CHOL and another study in which the same dose was administered for 28 days produced decreased levels of testicular cholesterol [66]. 

Another study in which 10 mg/100 g bw/day MSG was administered to adult rabbits for 21, 42 and 63 days indicated lower levels of CHOL compared to the Control group in each case [78]. In our study, we could not see the tendency of CHOL values to vary increasingly depending on MSG dose, but we observed the highest level of CHOL in group 4. Group 3 presented the lowest values, although no statistical differences were found. Similar results, with lower cholesterol values in the experimental groups than in the Control group, were also identified by Ogbuagu E. O. et al. (2019) [79]. In his study, the highest value is in the Control group, followed by the one with 1000 mg/kg bw/day, with a decrease to the maximum dose (1250 mg/kg bw/day). Also, comparing our results to the study of Nnadozie J. O. et al. (2019), an increase in CHOL values occurred compared to the Control group when they administered to adult rats the dose corresponding to the ADI dose in humans for 12 months [32].

#### 4.1.5. Triglycerides (TG)

TG characterises the body’s lipid profile, with high TG values indicating acute pancreatitis, liver and kidney damage or diabetes. An increase in TG can also be produced by exogenous substances [69,70,80]. Therefore, MSG administration is of interest, as the alleged liver damage showed that MSG could be responsible for elevated TG [81]. MSG could cause dysregulation of insulin levels, with hypertriglyceridemia as a consequence. Heavy consumption of MSG increases the levels of TNF-α. Accumulation of pro-inflammatory cytokines and chemokines in adipose tissue would play a key role in insulin resistance by obstructing inflammatory signalling and entry of immune cells into adipose tissue. Thus, changes in the levels of some adipocytokines have repercussions on insulin activity, with insulin resistance and an impairment of the lipid profile, by increasing lipid levels in the liver [44].

A possible dysregulation of the carbohydrate metabolism, manifested by insufficient insulin levels, can lead to increased lipolysis, with high production of fatty acids, possibly due to elevated levels of oxidative species. The esterification of fatty acids leads to TG, increasing lipid reserves in the liver and muscles [77]. Consequently, the hepatic synthesis of very low-density lipoprotein (VLDL) increases, with a blockage of chylomicron lipolysis. There is a decrease in the uptake of TG in peripheral tissues, and the remaining high TG are then transported to the liver [76,81,82]. At the same time, an increase in the bioavailability of fatty acids could promote lipid peroxidation [30].

In our study, the levels of TG increased as the MSG dose was higher, except in the Control group, which had the highest values after group 4. Although no statistical differences were observed, the group that received 6000 mg/kg bw/day presented the highest values of TG and CHOL, the parameters contouring the lipid profile of the subjects. Several experimental studies quantified the levels of TG after administration of different doses of MSG to groups of adult rats. Most of them observed increased levels of TG compared to the Control group: 2.4 g/kg bw/day, administered for 8 weeks [33]; 600 mg/kg bw/day, for 28 days (slightly increasing levels of TG) [61], 200 mg/kg bw/day, for 14 days [28], 50 mg/kg bw/day, for 4 weeks [57]; 4 mg/kg bw/day, for 7 days [42]; 10 mg/kg bw/day, for 30 and 60 days [36]; 0.5 g/kg bw/day and 1 g/kg bw/day for 2, respectively, 4 weeks (dose-proportional and time-proportional increase of TG levels) [41]. However, the scientific literature presents several studies with lower TG values in the experimental groups than in the Control group: 0.69 mg/g bw/day and 1.38 mg/g bw/day of MSG, for 28 days [58]; 4 mg/kg bw/day of MSG, for 28 days [66]; 4 mg/kg bw/day of MSG, for 14 days [38].

#### 4.1.6. Urea (UR) and Creatinine (CR)

Regarding renal damage, CR and UR are two suggestive parameters for evaluating possible changes at this level [67]. Since CR does not undergo tubular secretion or resorption, it is a relevant indicator of glomerular filtration rate and, implicitly, renal function [80]. 

According to scientific literature, MSG’s nephrotoxic effect is achieved through multiple mechanisms. Among these is the formation of free radicals at the renal level, considered a primary cause of the destruction at the cellular level [44]. The kidney is regarded as one of the organs with increased sensitivity to free radicals, possibly due to the abundance of long-chain unsaturated fatty acids in renal lipids. A decrease in CAT, SOD and glutathione-S-transferase levels and GSH activity may be a possible mechanism of MSG toxicity at this level. Low levels of GSH affect the balance between free radicals and endogenous antioxidant compounds. Also, elevated α-KG dehydrogenase activity or increased generation of free radicals following high intracellular calcium concentration may be other mechanisms of MSG toxicity [83].

UR is a biological compound with a crucial role in the body, as its synthesis contributes to the decrease of ammonia levels resulting from the catabolism of amino acids. Thus, the amount of this parameter is influenced by protein consumption. The detoxification process takes place through the biochemical reactions of the UR cycle [40,67,80,84]. UR is a valuable indicator in case of liver damage if it is lower than normal levels and for kidney damage, cardiac dysfunction, dehydration or a high-protein diet in case of high uraemia [84].

The specialised literature presents MSG as responsible for the dysregulation of tubular resorption, glomerular filtration rate and renal blood flow. All these factors can be causes of nephrotoxicity. These alterations result from oxidative stress production at this level [42]. The impairment of renal function produced by MSG could result from some of its metabolites’ effects on the kidney. Also, serum CR and UR levels increase when the kidney’s filtering capacity decreases [77].

In our study, CR levels do not present significant differences between the Control group and groups 1–3, with similar values. However, group 4 is distinguished, having the highest levels, with statistical differences compared to the Control group. Other experimental studies on adult rats support the idea that higher values of CR appear in the experimental group, compared to the Control one, when the following doses of MSG are administered as 3 g/kg bw/day for six months [83], and 15 mg/kg bw/day for 30 and 60 days, respectively (increased values depending on the period of administration) [27]. Also, the study by Al-Deri A. (2020) on 3–4-month-old adult rabbits concluded that a dose of 10 mg/100 g bw/day for 21, 42 and 63 days leads to increased values of CR compared to the Control group in each case. However, after 42 days of administration, CR levels were higher than after 63 days of MSG and almost reached the levels of CR after 21 days of MSG [78].

Also, several studies assessed the levels of CR an UR. They observed increased levels of both parameters compared to the Control group when administering MSG: 15 mg/kg bw/day, for 30 days [77]; the dose corresponding to the ADI dose in humans, for 12 months [32]; 4 g/kg bw/day, for 14 days [62]; 50 mg/kg bw/day, for 4 weeks [57], 4 mg/kg bw/day, for 7 days [42], 5 g/kg bw/day, for 30 days [35]; 500 mg/kg bw/day, 750 mg/kg bw/day, 1000 mg/kg bw/day and 1200 mg/kg bw/day, for 8 weeks—dose-dependent increase of CR levels/dose-dependent increase of UR levels, excepting the second group, which had the lowest UR values among the experimental groups [72]; 1.8 mg/kg bw/day, for 30 days [59]. In some studies, there was no correlation between the levels of CR and UR after MSG administration: 0.6 mg/g bw/day and 1.6 mg/g bw/day for 14 days with a dose-dependent increase of CR values and dose-dependent decrease of UR levels [67]; 8 mg/g bw/day, for 14 days with a decrease in the levels of CR and an increase of UR levels compared to the Control group [40]. In El-Sawy’s H. B. I. et al. (2018) study, a dose of 2 g/kg bw/day, administered for four weeks, produced a decrease in the levels of both CR and UR [29].

Regarding UR levels, in the present study, we have similar values for the Control group, groups 1 and 4, while group 3 presents the highest levels. The decreasing trend of values from group 3 to group 4 is also repeated for the UR, ALT, DB, TB and ALP parameters. Our hypothesis for this effect could sustain the body’s possible compensatory mechanisms. As we analysed the biochemical parameters, particularly valuable for liver and kidney function, we intended to complete the results with an anatomopathological evaluation of the hepatic and renal tissues. Also, as MSG was given orally, the stomach was subjected to anatomopathological analysis.

### 4.2. Histopathological Analysis

#### 4.2.1. Bile Duct Hyperplasia

Bile duct hyperplasia is considered to be a common background finding, being a spontaneous change in older animals, but it can also be induced or exacerbated by the treatment [48,85,86]. Minimal duct hyperplasia was found in all groups, especially in group 2, followed by the Control one. The fact that 75% of the Control group subjects suffer minimal duct hyperplasia prevents us from affirming that this modification is associated with MSG administration.

#### 4.2.2. Oval Cell Hyperplasia

Minimal oval cell hyperplasia was observed in the Control group and group 3, the experimental group having the highest values. Also, group 3 was the only group that presented moderate oval cell hyperplasia. Bile duct hyperplasia may be associated with oval cell hyperplasia [48]. Oval cell proliferation indicates a more substantial direct stimulus or reparative response to significant hepatobiliary injury. Significant injury to cholangiocytes or hepatocytes or inhibition of replication of mature hepatocytes may lead to oval cell proliferation [86]. Oval cell hyperplasia is most commonly observed in animals treated with hepatotoxic xenobiotics or in cases of infection, particularly with *Helicobacter hepaticus* and *Helicobacter bilis* [48,87].

#### 4.2.3. Mononuclear Cell Infiltrate

Minimal and moderate, mainly portal, multifocal mononuclear cell infiltrate was observed in all groups of our experiment. They are generally noticed in several chronic liver diseases of various aetiologies [88]. Regarding its pathogenesis, we can mention persistent noxious stimuli associated with infection, toxic xenobiotics, continued low-level parenchymal cell death and immune-mediated effects. Its diagnostic features may include bile-duct hyperplasia in some portal areas [48].

#### 4.2.4. Hepatic Macrovesicular and Microvesicular Steatosis

In our study, hepatic macrovesicular and microvesicular steatosis (mainly centrilobular) was observed only in the control group, while from group 2 to group 4, increasing percentages of minimal hepatic focal microvesicular steatosis were noticed. It can also be considered a physiological adaptation to an imbalance between the uptake of lipids from blood and the secretion of lipoproteins by the hepatocyte [48]. Nitrofurantoin, methotrexate, glucocorticoids, oestrogens or acetaminophen are a few drugs that could be associated with the production of macrovesicular steatosis [89].

#### 4.2.5. Other Hepatic Histological Alterations

Although many cases of cholangiocarcinoma are not related to a specific risk factor, primary hepatobiliary disease, genetic disorders, toxic exposures and infections could contribute to its development [90]. Cholangiocarcinoma with trans capsular infection occurred in 10% of subjects of group 1 and is rarely seen as spontaneous neoplasm in rats and mice but may occur following exposure to hepatotoxic xenobiotics [48].

The karyomegaly observed in group 4 occurs because of duplication of nuclear material in the absence of cytokinesis, reflecting hepatocyte polyploidy. Ageing rats of different strains have a predisposition to variations in cell size, nuclei and polyploidy [48,91]. Moderate diffuse lymphohistiocytic hepatitis and minimal portal fibrosis were observed only in group 3. Chronic cholangitis is characterised by chronic inflammation and fibrosis of the bile duct system, which reduces the bile duct calibre and obstruction [92]. Usually, cholangitis is caused by a bacterial infection, such as *Helicobacter* spp. [48]. In our study, chronic necro haemorrhagic focal cholangitis occurs only in the Control group. Eosinophilic focal cell alteration is noticed only in the Control group. Foci of cellular alteration are common in chronic (more than one year) experimental studies on rodents. Also, it may be noticed in short-duration toxicity studies if the subjects are exposed to certain xenobiotics [48].

#### 4.2.6. Hepatic Histological Modifications Produced by MSG in Rats Reported in the Scientific Literature

Scientific studies evaluating MSG toxicity on liver tissue reported several histological modifications following MSG administration, compared to the Control group, as follows: periportal hepatic necrosis associated with mononuclear cell infiltration and single-cell necrosis (2.4 g/kg bw of MSG, for 8 weeks) [33]; moderate atrophied, degeneration in the hepatocytes, marked necrosis, diffuse Kupffer cell proliferation in between some hepatocytes and surrounding the portal area (5 mg/kg bw of MSG, for 4 weeks) [37]; cytoplasmic fatty vacuolation and necrosis of the centrilobular hepatocytes, and fatty vacuolation of the interlobular hepatocytes was observed at a higher dose (5 mg/kg bw/day and 15 mg/kg bw/day of MSG, respectively, for 4 weeks) [26]; vacuolated cytoplasm, highly acidophilic cytoplasm, pyknosis, prominent Kupffer cells and dilated blood sinusoids (2 g/kg bw MSG, for 4 weeks) [25]; hepatocytes cell injury: hydropic degeneration, congested sinusoidal vessels and bi-nucleation (97 mg/kg bw MSG, for 6 weeks) [43]; dilatation of portal vein branches, vacuolar cytoplasmic degeneration of hepatocytes and inflammatory cell infiltrate within the liver lobule (120 mg/kg bw/day of MSG, for 3 months) [44]; marked dilatation of hepatic sinusoids, hepatocyte vacuolation with infiltration of inflammatory cells and small fragmented pyknotic nuclei and apoptotic hepatocytes (4 mg/kg bw/day of MSG, for 7 days) [42].

#### 4.2.7. Hyaline Droplets

Hyaline droplets represent low molecular weight protein accumulation within lysosomes caused by tubular reabsorption and hydrolysis balance disturbance. The reason for this imbalance is increased filtered protein loads or decreased catabolism [48]. Hyaline droplets occur regularly in younger male rats, but renal tubule epithelial cells of female and male and female mice do not typically contain them [93]. Diverse chemical substances can increase hyaline droplet levels [94]. In our study, hyaline droplets were observed mainly in group 3.

#### 4.2.8. Mineralisation

Mineralisation can occur spontaneously in laboratory animals or as a consequence of drug treatment. It is frequently observed in rodent toxicity studies. Mineralisation can also be a consequence of renal failure. More commonly, renal mineralization is found as a spontaneous lesion along the corticomedullary junction in rats as a background finding and is of no clinical consequence. Among rodents, female rats show a higher predisposition [48]. This modification was observed only in group 3.

#### 4.2.9. Cytoplasmic Vacuolisation

Cytoplasmic vacuolisation is characterised by intracellular accumulation of fluid, lipid, or other material within the epithelium. This results in a swollen, pale or granular cytoplasmic appearance. Although it may precede degeneration and necrosis, cytoplasmic vacuolisation may also indicate a reversible change or occur in normal animals [48]. Group 4 presented the highest values, expressed as a frequency of occurrence. Abd-Elkareem M. et al. (2022) [13] and Elbassuoni E. A. et al. (2018) also described vacuolisation in MSG-treated groups.

#### 4.2.10. Chronic Progressive Nephropathy

In our study, as CPN occurred in all groups, we attempted to grade it to evaluate if there is a correlation between its severity and MSG dose. Although there are no statistical differences between groups, we noticed a trend of increasing severity grade from the Control group to group 3, with a subsequent decrease of values in group 4.

CPN is a common, spontaneous, age-related disease of rodent kidneys, particularly in rats [95]. The disease appears in the commonly used strains of laboratory rats [96]. CPN has been described as a degenerative to atrophic disease with compensatory hypertrophy and hyperplasia [95]. The aetiology of chronic progressive nephropathy of ageing is unknown. Genetic factors appear important because non-albino strains are less affected than albino strains [97]. Sprague-Dawley and F344 generally have an onset and higher incidence and severity than the Wistar, Brown Norway and Long-Evans rat strains. Also, no human renal disease is similar to CPN [95].

Furthermore, the disease appears more severe in male than female rats. In addition to strain, age and sex differences, hormones, diet and microflora are other factors that can modulate the incidence and severity of CPN-associated lesions [95,97]. A possible contributing factor may be the high protein level of the male rat kidney [96]. Thus, a low-protein diet could be protective because reducing caloric intake reduces disease incidence and severity while increasing dietary protein has a contrary effect [96,97]. In addition to dietary protein, carbohydrates, sodium excess or amino acid toxicity also influence the disease. However, although factors such as genetic background, sex and diet affect the evolution of this condition, they do not appear to directly determine the initiation of this ageing-associated renal disease [97]. There is also evidence that xenobiotic treatment may aggravate or exacerbate this condition [50]. The attention was drawn to five chemicals in particular—anthraquinone, benzofuran, *tert*-butyl alcohol, ethyl benzene and tetrafluoroethylene.

Serum urea nitrogen and creatinine levels are not significantly elevated until the advanced stages of the disease. Advanced CPN is a significant factor underlying the spontaneous development of atypical tubule hyperplasia [96].

#### 4.2.11. Renal Histological Modifications Produced by MSG in Rats Reported in the Scientific Literature

Some of the renal histological alterations following MSG consumption observed in experimental studies on rats and mice, compared to the Control group, are presented as follows: vascular congestion, interstitial inflammatory infiltrate, hyaline casts and cellular casts in the renal tubules (120 mg/kg bw/day of MSG, for 3 months) [44]; dilatation and necrosis and fatty change of renal tubules was noticed at a higher dose (5 mg/kg bw/day and 15 mg/kg bw/day MSG, respectively, for 4 weeks) [26]; marked swelling in the glomeruli, decreased Bowman’s spaces, vacuolar degeneration in addition to infiltration of inflammatory cells in the interstitial tissue (4 mg/kg bw/day of MSG, for 7 days) [42]; increase in Bowman’s space, congestion of glomerular blood vessels and beginning of mesangial cells proliferation (15 mg/kg bw MSG, for 30 days) and a high increase in Bowman’s space, mitosis in mesangial cells, congested capillaries and congestion and haemorrhage of blood vessels (15 mg/kg bw MSG, for 30 days) [27].

#### 4.2.12. Other Observations

No histological alterations were observed among the subjects after stomach evaluation.

Polyuria and polydipsia were noted to be more pronounced with increasing MSG dose, but in the case of group 4, they were very marked compared to the other groups. One possible explanation is the high amount of sodium ions in MSG.

## 5. The Hypothesis of the Development of a Defence Mechanism Triggered by MSG Sub-Chronic Consumption in Ageing Rats

In this study, we attempted to explain our results based on the hormesis phenomenon and the complex biochemical implications of GLA, also considering the advanced age of the rats.

Hormesis is a dose–response relationship when a low-dose effect is opposite to the one observed at high doses. It can explain how cells respond to low-dose exposure to stressors [98]. The adaptative beneficial effect on the organism of low doses of exposure to a chemical substance or environmental factor characterises the phenomenon. In contrast, high doses can be toxic [98,99]. The mechanism of hormesis as an adaptive response is based on the idea that hormetic chemicals disrupt normal cellular function [100]. Living cells can react to environmental signals and change the rules of linear relationships [98]. Also, hormetic physiological responses are not the manifestation of a specific single signalling cascade. They require the coordination of multiple cellular processes. Hormetic responses have been described under several terms, such as biphasic, bidirectional, dual, opposite, paradoxical or atypical effects [100]. During the hormesis response, the organism reacts to a low dose of toxic substances characteristically, resulting in an inverted U-shaped or J-shaped dose–response curve [99,100]. Since this phenomenon is closely related to oxidative chemical substances and considering the approach that MSG is responsible for oxidative stress production, we think that there may be a connection between them. There are also studies, such as the one of Hernández-Bautista R. J. et al. (2014) on MSG-induced obese ageing mice, approached from the perspective of hormesis [101,102]. Due to the fact that statistically significant differences between the groups can be observed only in a few cases (ALT: group 3 versus group 4; ALP: Control group versus group 4 and group 1 versus group 4; CR: Control group versus group 4), the hormesis phenomenon could be an apparent one and the observed differences in the responses are attributable to the unequal and small group size, heterogeneity among animals in susceptibility to MSG effects and other factors listed in Section 6.

Therefore, low-level ROS might activate different transcription factors sensitive to redox status changes, such as nuclear factor erythroid 2–related factor (Nrf2) [99]. In response to modifications in the cellular redox state, Nrf2 is released from its repressor Kelch-like ECH-associated protein 1(Keap-1), phosphorylated and translocated into the nucleus. There, it binds to the antioxidant response element (ARE), induces antioxidant and phase II detoxifying enzyme expression and increases GSH content. This mechanism is influenced by several factors, such as health, individual genetic background or age. An imbalanced redox state, with weaker antioxidant systems and increased ROS production, is characteristic of ageing animals. Even though they may present organ alterations due to advanced age, the stressors may strengthen the organism, increasing the survival capacity [101].

Although the inductor hormetic dose is difficult to establish and the hormetic mechanisms are probably more complex, with more responses activated upon oxidative stress, we attempted to bring this approach to our study. A decrease (even slightly) in the levels of some biochemical/metabolic parameters from the Control group to group 1, or even to groups 2 and 3, in some cases, may be due to the hormesis effect. This aspect can be observed in the case of AST, CHOL, TG, UR, DB and TB. In this case, low doses of MSG may have a protective effect in ageing rats. In the case of DB and TB, we may talk about an inverted U-shaped dose–response curve with the highest values in group 2. In the case of TG, we may consider a J-shaped dose–response curve, describing low-dose reduction and high-dose enhancement of adverse effects.

Also, the biochemical implications of GLA may explain the defence mechanism triggered by sub-chronic MSG consumption. GLA is the primary nitrogen collector in the organism, transporting nitrogen from the tissues to the liver and kidneys for ureagenesis [103]. Intestinal transformation of α-KG into GLU under the action of gut bacteria is an essential source of the amino acid. However, as the host ages, the gut bacteria are lost, with a decreased ability to synthesise GLA [104]. Also, the alanine-glucose cycle involves the transport of tissue nitrogen to the liver as alanine, obtained by pyruvate- and GLA-dependent ALT transamination reaction [105]. The variability of serum ALT and AST values at the NOAEL dose may be the effect of the age of the subjects and the size of the group or the different reactivity of rats to relatively high amounts of MSG. Reactions catalysed by transaminases are reversible—the reaction equilibrium shifts towards GLA synthesis in the tissues and α-KG in the liver. The decrease in ALT/AST values between group 3 and group 4 can be attributed to the saturation of enzymes with the substrate, as the substrate may function as an allosteric modulator. Another factor to consider in transamination reactions is the age-dependent metabolic status of vitamin B (pyridoxine), which is an active participant in the transfer of the amino group as pyridoxal 5′-phosphate (PLP) [106].

The key enzyme in CHOL synthesis is NADPH-dependent HMG-CoA reductase. NADPH is the coenzyme of glutathione reductase and aldose reductase, the key enzyme in glucose metabolism via the polyol pathway. Both of them are marker enzymes in oxidative stress [107]. Increased levels of oxidative stress caused by MSG administration may lead to NADPH depletion and decreased HMG-CoA reductase activity, with reduced cholesterolemia, as observed in group 3. However, according to the scientific literature data, the increasing tendency of serum CHOL levels at higher doses of MSG can be attributed to profound cellular changes in the redox balance or to mitochondrial dysfunction.

TG are synthesised de novo in the liver, using intermediates of carbohydrate metabolism: acetyl-CoA as a precursor for fatty acid synthesis and dioxyacetone phosphate to obtain glycerol-3P (glycerol-3 phosphate). In the case of a calorie-balanced diet, acetyl-CoA will be oxidised in the Krebs cycle to CO_2_, generating reduced coenzymes (NADH and FADH_2_). The normal functioning of the Krebs cycle depends on the presence of both precursors, acetyl-CoA and oxaloacetate (OA), in equimolecular quantities. Insulin resistance in aged animals can alter Krebs’ cycle function, with an OA/acetyl-CoA ratio < 1, with serum hypertriglyceridemia as a consequence [108]. MSG administration and the series of biochemical transformations: GLA → α-KG → succinyl-CoA → succinate → fumarate → malate → OA may be an anaplerotic reaction of the Krebs cycle and supply the mitochondria with OA. This aspect would explain the lower TG values in groups 1, 2 and 3 compared to the Control group. However, at high doses (group 4), MSG produces mitochondrial dysfunction, with Krebs cycle damage loss of electrons at the respiratory chain level, the main cause of ROS production.

## 6. Limitations of the Study

Although we tried to conduct the study in optimal conditions, we identified certain limiting aspects. However, some of these limitations can be overcome in the future, leading to valuable results. The limitations of this study are presented as follows:

- Logistical difficulties prevented us from conducting a parallel study on younger adult Wistar rats to compare MSG’s effect on different age groups. We could obtain valuable results through this approach, which would be a future research direction we propose. Also, a higher number of subjects in each group would have been indicated for better precision of the results.
- Also, due to logistical difficulties, it was not possible to examine the subjects who died before the end of the administration of the entire quantity of MSG. Therefore, some possible toxic effects of MSG that could have caused death were omitted.
- Extrapolating the results obtained on experimental animals to the human species is quite challenging. Achieving highly relevant data in this regard would require clinical trials.
- The self-administration method of MSG used in the study has advantages and disadvantages. Its limitations include the lower dosage accuracy than oral gavage or parenteral methods. Also, it relies on the natural circadian timing of alimentation and depends on individual preferences for flavours, palatability issues and changes in behaviour over time.
- Selecting the MSG doses administered within the groups to be relevant for the human species was difficult. The heterogeneity of doses administered in similar scientific studies contributed to this aspect.

## 7. Conclusions

MSG is a controversial chemical substance, as both the advantages and disadvantages of its use are approached in the scientific literature. Despite the positive results of experimental studies regarding the toxicity of MSG at different levels, the regulatory authorities in food safety (EFSA, FDA) do not question the safety of using MSG in food. Also, GLA metabolic pathways support its utility for the human body. The organism behaviour at ADI and NOAEL doses of MSG and how MSG toxicity is reflected in subjects potentially affected by advanced age was assessed using two-year-old Wistar rats.

After evaluating the biochemical parameters, we can conclude that, even though, in most cases, there were no statistical differences in the levels of the parameters between the groups, there are some changes that could have biological importance. This aspect refers to ALT, ALP, DB, TB and UR, which are lower at high doses. Also, a decrease of AST, CHOL, TG, UR, DB and TB from the Control group to group 1, or even to groups 2 and 3, in some cases, could be observed. The hormesis effect, with low-level ROS protective effects and the biochemical implications of GLA, may represent the hypothesis of a potential defence mechanism triggered by MSG sub-chronic consumption in ageing rats.

Among the experimental studies discussed in this article are those in which MSG toxicity is evaluated for a shorter administration period, included in the acute toxicity category. However, we could observe that even though smaller doses of the additive were administered for a shorter time than ours, MSG proved to produce pathological changes. This aspect is somewhat confusing because, according to our results, we cannot say that MSG was precisely harmful. On the contrary, we can affirm that, after the sub-chronic administration of MSG, the positive aspects of GLA are better reflected than the negative ones in ageing rats and could also be relevant for the elderly human population. This aspect is consistent with the fact that the regulatory authorities accept MSG in food safety, and it is widely used.

Still, even considering all these aspects, it is not simple to formulate a conclusion regarding the acceptance limits of MSG among rats, even more so in the human species. A more profound approach to the subject is necessary to assess MSG sub-chronic consumption’s risk/benefit ratio.

## Figures and Tables

**Table 1:** Preclinical studies regarding the toxicity of MSG conducted on adult rats.

Year	Tested Animals	Animals/Group	Daily MSG Dose/kg bw	Study Duration	Tested Parameters	Results	Ref.
2021	Male rats	10/group	15 mg	30 days and 60 days	Kidney tissue	Proliferated and enlarged mesangial cells	[27]
					Serum CR	Increased levels	
2020	Male Wistar rats	10/group	2.4 g	8 weeks	Liver tissue	Periportal hepatic necrosis; mononuclear cell infiltration	[33]
					GSH, GST, SOD, CAT *	Decreased levels	
					AST, ALT, ALP, TB, GGT, CHOL, TG **	Increased levels	
2019	Male Albino Rats	12/group	97 mg	6 weeks	Liver tissue	Hydropic degeneration; congested sinusoidal vessels; binucleation	[43]
					SOD, CAT, GSH, GPx ***	Decreased levels	
					ALT, AST, ALP, Bilirubin	Increased levels	
2021	Male rats	10/group	120 mg	3 months	Liver and kidney tissues	Dilatation of portal veins; vacuolar cytoplasmic degeneration of hepatocytes; hyaline and cellular casts in the renal tubules	[44]
					Total antioxidant capacity	Decreased levels	
					AST, ALT, UR, CR	Increased levels	
2022	Male rats	10/group	10 mg	4 weeks	CHOL, TG, LDL **, AST, ALT	Increased levels	[36]
					HDL **	Decreased levels	
				8 weeks	CHOL, TG, LDL, AST, ALT	Increased levels	
					HDL	Decreased levels	
2020	Virgin female Wistar rats	7/group	200 mg	14 days (acute toxicity)	Progesterone, Oestrogen	Increased levels	[28]
					TG, CHOL	Increased levels	
					Ovarian tissue	Moderate to severe deposits of collagen tissue with fibrosis were observed in the ovarian stroma	
2017	Male rats	10/group	5 g	30 days	AST, ALT, ALP, UR, CR	Increased levels	[35]
2014	Wistar rats	6/group	3 g	7 days (acute toxicity)	Stomach tissue	Multiple congested blood vessels in the gastric submucosa	[45]
			6 g			Increase of connective tissues lamina propria and around the basal parts of the gastric gland	

bw = body weight; * GSH = Glutathione; GST = Glutathione S transferase; SOD = superoxide dismutase; CAT = Catalase; ** AST = Aspartate aminotransferase; ALT = Alanine aminotransferase; ALP = Alkaline phosphatase; TB = Total bilirubin; GGT = Gamma-glutamyltransferase; CHOL= Cholesterol; TG = Triglycerides; CR = Creatinine; UR = Urea; LDL = low-density lipoprotein; HDL = high-density lipoprotein; *** GPx = Glutathione peroxidase.

**Table 2:** Amounts of MSG/per group administered in the experimental animal model (two-year-old rats).

Group	Dose	Observations
1	185 mg MSG/kg bw/day	Approximately equal to the maximum daily dose of MSG in the human species—30 mg/kg bw per day according to EFSA [9]
2	1500 mg MSG/kg bw/day	Half of the third group dose
3	3000 mg MSG/kg bw/day	A similar value to the NOAEL dose for MSG −3200 mg/kg bw according to EFSA [9]
4	6000 mg MSG/kg bw/day	Twice the approximate NOAEL dose
Control	MSG non-consumer group	-

**Table 3:** Analysis methods and the specific reagent for each parameter.

Biochemical/ Metabolic Parameter	Analysis Method	Reagent
Aspartate Aminotransferase (AST)	IFCC (International Federation for Clinical Chemistry) standardised kinetics with the pyridoxal phosphate method	Aspartate aminotransferase, Roche Diagnostics, GmbH, Mannheim, Germany (ASTL)
Alanine Aminotransferase (ALT)	IFCC (International Federation for Clinical Chemistry) standardised kinetics with the pyridoxal phosphate method	Alanine aminotransferase, Roche Diagnostics, GmbH, Mannheim, Germany (ALTL)
Alkaline phosphatase (ALP)	Spectrophotometric method (colourimetric test)	Alkaline phosphatase IFCC, 2nd generation, Roche Diagnostics, GmbH, Mannheim, Germany (ALP2)
Total bilirubin (TB)	Spectrophotometric (colourimetric) method	Total bilirubin DPD, 2nd generation, Roche Diagnostics, GmbH, Mannheim, Germany (BILT2)
Direct bilirubin (DB)	Spectrophotometric (colourimetric) method	Direct bilirubin, 2nd generation, Roche Diagnostics, GmbH, Mannheim, Germany (BILD2)
Total cholesterol (CHOL)	Spectrophotometric (enzymatic-colourimetric) method	Cholesterol Roche Diagnostics, GmbH, Mannheim, Germany (CHOL2)
Triglycerides (TG)	Spectrophotometric (enzymatic-colourimetric) method	Triglycerides Roche Diagnostics, GmbH, Mannheim, Germany (TRIGL)
Creatinine (CR)	Kinetic (enzymatic-colourimetric) Jaffé method	Creatinine Jaffé, 2nd generation, Roche Diagnostics, GmbH, Mannheim, Germany (CREJ2)
Urea (UR)	Spectrophotometric (kinetic) method	Urea, Roche Diagnostics, GmbH, Mannheim, Germany (Ureal)

**Table 4:** Histopathological modifications of hepatic tissue measured after three months of daily MSG administration in two-year-old rats (bw—body weight).

Histopathological Modification	Occurrence Frequency of Modification/Group *				
	Control Group (*n* ^†^ = 12)	Group 1 (*n* = 10)	Group 2 (*n* = 9)	Group 3 (*n* = 7)	Group 4 (*n* = 12)
Bile duct hyperplasia (minimal)	75% (9/12)	30% (3/10)	77.77% (7/9)	42.86% (3/7)	58.33% (7/12)
Bile duct hyperplasia (moderate)	-	10% (1/10)	22.22% (2/9)	42.86% (3/7)	16.66% (2/12)
Oval cell hyperplasia (minimal)	33.33% (4/12)	-	-	57.14% (4/7)	-
Oval cell hyperplasia (moderate)	-	-	-	14.29% (1/7)	-
Focal necrotic hepatitis (minimal)	8.33% (1/12)	-	-	-	-
Multifocal mononuclear cell infiltrate (mainly portal—minimal)	33.33% (4/12)	70% (7/10)	66.66% (6/9)	42.86% (3/7)	66.66% (8/12)
Multifocal mononuclear cell infiltrate (mainly portal—moderate)	-	-	22.22% (2/9)	14.29% (1/7)	-
Focal chronic necro haemorrhagic cholangitis	8.33% (1/12)	-	-	-	-
Hepatic macro- and micro-vesicular steatosis (mainly centrilobular)	8.33% (1/12)	-	-	-	-
Hepatic focal microvesicular steatosis (minimal)	-	-	11.11% (1/9)	14.29% (1/7)	16.66% (2/12)
Focal hydropic change (minimal)	8.33% (1/12)	-	-	-	-
Glycogenosis (glycogen storage—minimal)	8.33% (1/12)	10% (1/10)	-	-	-
Eosinophlic focal cell alteration	8.33% (1/12)	-	-	-	-
Diffuse lymphohistiocytic hepatitis (moderate) and portal fibrosis (minimal)	-	-	-	14.29% (1/7)	-
Individual hepatocytic death (apoptosis/necrosis)	-	-	-	14.29% (1/7)	-
Cholangiocarcinoma	-	10% (1/10)	-	-	-
Karyomegaly	-	-	-	-	16.66% (2/12)

* Expressed as a percentage of the total number of the subjects/group; ^†^ number of subjects/group.

**Table 5:** Histopathological modifications of renal tissue measured after three months of daily MSG administration in two-year-old rats (bw—body weight).

Histopathological Modifications	Occurrence Frequency of Modification/Group *				
	Control Group (*n* ^†^ = 12)	Group 1 (*n* = 10)	Group 2 (*n* = 9)	Group 3 (*n* = 7)	Group 4 (*n* = 12)
Hyaline droplet accumulation	8.33% (1/12)	-	-	42.86% (3/7)	8.33% (1/12)
Tubular pigment	58.33% (7/12)	80.00% (8/10)	33.33% (3/9)	71.43% (5/7)	58.33% (7/12)
Mineralisation	-	-	-	14.29% (1/7)	-
Cytoplasmic vacuolisation	8.33% (1/12)	-	33.33% (3/9)	42.86% (3/7)	58.33% (7/12)
Chronic progressive nephropathy	75.00% (9/12)	80.00% (8/10)	77.77% (7/9)	85.71% (6/7)	91.66% (11/12)

* Expressed as a percentage of the total number of the subjects/group; ^†^ number of subjects/group.

## Data Availability

Not applicable.

## Supplementary Materials

The following supporting information can be downloaded at https://www.mdpi.com/article/10.3390/nu15204436/s1, Table S1: AST and ALT values measured after three months of daily MSG administration in two-year-old rats (bw—body weight); Table S2: ALP values measured after three months of daily MSG administration in two-year-old rats (bw—body weight); Table S3: Direct bilirubin (DB) and total bilirubin (TB) values measured after three months of daily MSG administration in two-year-old rats (bw—body weight); Table S4: Total cholesterol (CHOL) values measured after three months of daily MSG administration in two-year-old rats (bw—body weight); Table S5: Triglyceride (TG) values measured after three months of daily MSG administration in two-year-old rats (bw—body weight); Table S6: Creatinine (CR) values measured after three months of daily MSG administration in two-year-old rats (bw—body weight); Table S7. Urea (UR) values were measured after three months of daily MSG administration in two-year-old rats (bw—body weight); Table S8. Chronic progressive nephropathy grade measured after three months of daily MSG administration in two-year-old rats (bw—body weight).
