# Supplementary material for: Potential Defence Mechanisms Triggered by Monosodium Glutamate Sub-Chronic Consumption in Two-Year-Old Wistar Rats
Source: Nutrients. 2023 Oct 19;15(20):4436. doi: 10.3390/nu15204436 (PMC10610236; doi:10.3390/nu15204436)
Supplement: Supplementary file 1 [file nutrients-15-04436-s001.zip › nutrients-2645633-supplementary.pdf]

Potential Defence Mechanisms Triggered by Monosodium Glutamate Sub-Chronic Consumption in Two-Year-Old Wistar Rats

Octavia-Laura Moldovan [1,*], Camil-Eugen Vari [2], Amelia Tero-Vescan [3], Ovidiu Simion Cotoi [4,5], Iuliu Gabriel Cocuz [4,5], Flaviu Alexandru Tabaran [6], Romelia Pop [6], Ibolya Fülöp [7], Rafael Florin Chis [8], Ioana-Andreea Lungu [1] and Aura Rusu [9]

[1] Medicine and Pharmacy Doctoral School, George Emil Palade University of Medicine, Pharmacy, Science, and Technology of Targu Mures, 540142, Targu Mures, Romania;
[2] Pharmacology and Clinical Pharmacy Department, Faculty of Pharmacy, George Emil Palade University of Medicine, Pharmacy, Science, and Technology of Targu Mures, 540142 Targu Mures, Romania;
[3] Medical Chemistry and Biochemistry Department, Faculty of Medicine in English, George Emil Palade University of Medicine, Pharmacy, Science, and Technology of Targu Mures, 540142 Targu Mures, Romania;
[4] Pathophysiology Department, Faculty of Medicine, George Emil Palade University of Medicine, Pharmacy, Science, and Technology of Targu Mures, 540142 Targu Mures, Romania;
[5] Pathology Department, Mures Clinical County Hospital, 540011, Targu Mures, Romania
[6] Department of Pathology, Faculty of Veterinary Medicine, University of Agricultural Sciences and Veterinary Medicine of Cluj-Napoca, Calea Manastur, 400372 Cluj-Napoca, Romania.)
[7] Toxicology and Biopharmacy Department, Faculty of Pharmacy, George Emil Palade University of Medicine, Pharmacy, Science, and Technology of Targu Mures, 540142 Targu Mures, Romania;
[8] George Emil Palade University of Medicine, Pharmacy, Science, and Technology of Targu Mures, 540142, Targu Mures, Romania;
[9] Pharmaceutical and Therapeutic Chemistry Department, Faculty of Pharmacy, George Emil Palade University of Medicine, Pharmacy, Science, and Technology of Targu Mures, 540142 Targu Mures, Romania
* Correspondence: octavia.moldovan@umfst.ro (O.-L.M.)

SUPPLEMENTARY MATERIALS

Table S1. AST and ALT values measured after three months of daily MSG administration in two-years-old rats (bw – body weight).

Group	No. of subjects/ group	MSG dose	AST (U/l) (Mean ± standard deviation)	ALT (U/l) (Mean ± standard deviation)
Control	12	Non-consumer	148 ± 63.3	47.5 ± 12.9
1	10	185 mg/kg bw	115 ± 55.3	50.2 ± 18.2
2	9	1500 mg/kg bw	135 ± 46.6	47.1 ± 13
3	7	3000 mg/kg bw	119 ± 113	*62.1± 21.2
4	12	6000 mg/kg bw	120 ± 40.9	*41.9 ± 10.3

Values represent the mean ± SD. One-way ANOVA, post hoc Dunnett's, and Tukey's tests were performed ($p \leq 0.05$). Statistical differences were found between groups 3 and 4 for the ALT enzyme (statistical differences being marked with *).

Table S2. ALP values measured after three months of daily MSG administration in two-year-old rats (bw – body weight).

Group	No. of subjects/ group	MSG dose	ALP (U/l) (Mean ± standard deviation)
Control	12	Non-consumer	57.7 ± 28.2
1	10	185 mg/kg bw	*65.7 ± 19.6
2	9	1500 mg/kg bw	39.8 ± 18.8
3	7	3000 mg/kg bw	51.6 ± 18.9
4	12	6000 mg/kg bw	*26.5 ± 8.68

Values represent the mean ± SD. One-way ANOVA, post hoc Dunnett's, and Tukey's tests were performed ($p \leq 0.05$). Statistical differences exist between group 4 and the Control group and between group 1 and group 4 (statistical differences being marked with *).

Table S3. Direct bilirubin (DB) and total bilirubin (TB) values measured after three months of daily MSG administration in two-year-old rats (bw – body weight).

Group	No. of subjects/ group	MSG dose	DB (mg/dl) (Mean ± standard deviation)	TB (mg/dl) (Mean ± standard deviation)
Control	12	Non-consumer	0.0592 ± 0.0358	0.0717 ± 0.0369
1	10	185 mg/kg bw	0.058 ± 0.0336	0.067 ± 0.0424
2	9	1500 mg/kg bw	0.0775 ± 0.0139	0.0878 ± 0.0393
3	7	3000 mg/kg bw	0.0625 ± 0.0191	0.0863 ± 0.025
4	12	6000 mg/kg bw	0.055 ± 0.0109	0.0808 ± 0.0281

Values represent the mean ± SD. One-way ANOVA, post hoc Dunnett's, and Tukey's tests were performed ($p \leq 0.05$).

Table S4. Total cholesterol (CHOL) values measured after three months of daily MSG administration in two-year-old rats (bw – body weight).

Group	No. of subjects/ group	MSG dose	CHOL (mg/dl) (Mean ± standard deviation)
Control	12	Non-consumer	143 ± 91
1	10	185 mg/kg bw	131 ± 48.9
2	9	1500 mg/kg bw	146 ± 74
3	7	3000 mg/kg bw	116 ± 33.2
4	12	6000 mg/kg bw	154 ± 71

Values represent the mean ± SD. One-way ANOVA, post hoc Dunnett's, and Tukey's tests were performed ($p \leq 0.05$).

Table S5. Triglyceride (TG) values measured after three months of daily MSG administration in two-year-old rats (bw – body weight).

Group	No. of subjects/ group	MSG dose	TG (mg/dl) (Mean ± standard deviation)
Control	12	Non-consumer	181 ± 166
1	10	185 mg/kg bw	127 ± 39.3
2	9	1500 mg/kg bw	143 ± 55.5
3	7	3000 mg/kg bw	155 ± 125
4	12	6000 mg/kg bw	253 ± 141

Values represent the mean ± SD. One-way ANOVA, post hoc Dunnett's, and Tukey's tests were performed ($p \leq 0.05$).

Table S6. Creatinine (CR) values measured after three months of daily MSG administration in two-years-old rats (bw – body weight).

Group	No. of subjects/ group	MSG dose	CR (mg/dl) (Mean ± standard deviation)
Control	12	Non-consumer	0.346 ± 0.128
1	10	185 mg/kg bw	0.36 ± 0.13
2	9	1500 mg/kg bw	0.399 ± 0.121
3	7	3000 mg/kg bw	0.379 ± 0.0976
4	12	6000 mg/kg bw	*0.485 ± 0.146

Values represent the mean ± SD. One-way ANOVA, post hoc Dunnett's, and Tukey's tests were performed ($p \leq 0.05$). Statistical difference was observed between the Control group and group 4 (statistical differences being marked with *).

Table S7. Urea (UR) values measured after three months of daily MSG administration in two-year-old rats (bw – body weight).

Group	No. of subjects/ group	MSG dose	UR (mg/dl) (Mean ± standard deviation)
Control	12	Non-consumer	44.7 ± 15.4
1	10	185 mg/kg bw	42.9 ± 7.88
2	9	1500 mg/kg bw	38.7 ± 6.85
3	7	3000 mg/kg bw	49.6 ± 12.6
4	12	6000 mg/kg bw	43.6 ± 5.61

Values represent the mean ± SD. One-way ANOVA, post hoc Dunnett's, and Tukey's tests were performed ($p \leq 0.05$).

Table S8. Chronic progressive nephropathy grade measured after three months of daily MSG administration in two-year-old rats (bw – body weight).

Group	No. of subjects/ group	MSG dose	CPN grade (Mean ± standard deviation)
Control	12	Non-consumer	2.25 ± 2.05
1	10	185 mg/kg bw	2.50 ± 2.27
2	9	1500 mg/kg bw	2.67 ± 2.50
3	7	3000 mg/kg bw	3.57 ± 2.37
4	12	6000 mg/kg bw	3.42 ± 2.61

Values represent the mean ± SD. One-way ANOVA, post hoc Dunnett's, and Tukey's tests were performed ($p \leq 0.05$).
